# Increasing the Particle Size and Magnetic Property of Iron Oxide Nanoparticles through a Segregated Nucleation and Growth Process

**DOI:** 10.3390/nano14100827

**Published:** 2024-05-09

**Authors:** Yiyang Liu, Sheng Wang, Qin Wang, Liping Wang, Jianghui Dong, Baolin Zhang

**Affiliations:** 1 School of Materials Science and Engineering, Guilin University of Technology, Guilin 541004, China; 1020210185@glut.edu.cn; 2 School of Intelligent Medicine and Biotechnology, Guilin Medical University, Guilin 541004, China; qinwang997@163.com (Q.W.); liping.wang@mymail.unisa.edu.au (L.W.); jianghui.dong@mymail.unisa.edu.au (J.D.)

**Keywords:** iron oxide nanoparticles, methods of adding reaction materials, nanoparticle size, nucleation and growth separation, saturation magnetization

## Abstract

Iron oxide nanoparticles (IONs) with good water dispersibility were prepared by the thermal decomposition of iron acetylacetonate (Fe(acac)_3_) in the high-boiling organic solvent polyethylene glycol (PEG) using polyethyleneimine (PEI) as a modifier. The nucleation and growth processes of the crystals were separated during the reaction process by batch additions of the reaction material, which could inhibit the nucleation but maintain the crystal growth, and products with larger particle sizes and high saturation magnetization were obtained. The method of batch addition of the reactant prepared IONs with the largest particle size and the highest saturation magnetization compared with IONs reported using PEG as the reaction solvent. The IONs prepared by this method also retained good water dispersibility. Therefore, these IONs are potentially suitable for the magnetic separation of cells, proteins, or nucleic acids when large magnetic responses are needed.

## 1. Introduction

Nanomaterials have been continuously developed and improved for their usage in various fields, including energy [1], the environment [2], information [3], medicine [4,5,6,7,8], and others. Nanoparticles match the size of many biological macromolecules such as nucleic acids and proteins [9]. In addition, magnetic nanoparticles possess unique magnetic properties that mean their movement is controllable under the action of an applied magnetic field [9,10]. Therefore, they have a broad application prospect in the biomedical field. For in vivo applications, magnetic nanoparticles can not only be used as markers to assist in the detection and diagnosis of clinical pathology [11,12], but also as a tool for loading certain drugs to achieve directional transport and release [13,14]. For in vitro applications, the surface of magnetic nanoparticles can be modified with a variety of ligands (or receptors), which can be specifically bound to the receptors (or ligands) for the detection and separation of specific cells, viruses, proteins, and nucleic acids under the action of an applied magnetic field [15,16,17,18].

IONs synthesized by thermal decomposition have the advantages of high magnetization and good dispersion, making them suitable as a medium for magnetic separation. The common organic compound precursors used for the synthesis of IONs by thermal decomposition are iron pentacarbonyl, iron acetylacetonate, metal complexes of oleic acid, etc. And the commonly used solvents are oleic acid, oleylamine, 1-octadecene, imidazolium ionic liquids, and different molecular weight polyethylene glycols. IONs prepared with oleic acid, oleylamine, 1-octadecene, imidazolium ionic liquid as solvents or modifiers are generally non-water-soluble, and need to be dispersed in weakly polar or non-polar organic solvents [19,20] such as hexane, toluene and so on. To achieve good dispersion of IONs synthesized using these solvents in water, it is generally necessary to use water-soluble surfactants [21]. PEG is a non-toxic, hydrophilic solvent with good biocompatibility [22]. IONs prepared using PEG as a solvent, PEI as a modifier and iron acetylacetonate as a precursor exhibit good water dispersibility [23].

The high magnetic requirement of IONs in the process of magnetic separation imposes stringent demands on their particle size [24,25]. Generally, larger particle sizes correspond to better magnetic properties [26]. Several methods have been proposed for the control of the particle sizes of non-aqueous dispersed IONs. For instance, using an iron oleate complex as the precursor and 1-octadecene as the solvent, IONs with sizes ranging from 9 to 14 nm can be prepared by adjusting the temperature and reaction time [27]. Additionally, employing the seed-mediated method allows for the preparation of larger crystals [21]. However, there are fewer methods available for increasing the particle size of water-dispersible, especially PEG-coated, IONs. Additionally, the saturation magnetization of IONs synthesized with PEG as solvent and modifier is generally below 60 emu/g at 300 K [23]. It was observed that increasing the temperature and reaction time only marginally increased the particle size of these water-dispersible nanoparticles [28]. In fact, we also attempted to use the seed-mediated method to further increase the size of these PEG-modified water-dispersible nanoparticles, but found only a limited increase in the sizes of the particles.

The nucleation and growth of crystals is a complex process. The current theories used to describe the nucleation process are mainly the LaMer classical nucleation–diffusion model [29] and the non-classical two-step nucleation mechanism [30]. The classical nucleation theory posits that monomers in solution aggregate and nucleate when the degree of supersaturation surpasses a threshold sufficient to overcome the nucleation energy barrier. Subsequently, the monomers diffuse to the surface of the nucleus and merge with the crystal structure. The non-classical nucleation theory suggests that monomers form sub-stable dense liquid-phase clusters before nucleation, which nucleate with a greater energy advantage than monomer nucleation. Moreover, it proposes that the smallest growing unit for growth is not the monomer, as stated in classical theory, but rather the sub-stable dense clusters or even nanoparticles [31,32]. Both the classical nucleation theory and non-classical nucleation theory regarding crystal nucleation and growth can be summarized in three steps: 1. nucleation; 2. the diffusion of the growth unit; 3. growth unit merging and crystal growth. Generally, in the synthesis process of nanoparticles, the raw materials are added at once; adding a large amount of raw material at the beginning of the reaction leads to excessive supersaturation, causing numerous nucleation to occur and resulting in the generation of a large number of grains subsequently but with limited particle sizes.

Based on this, the present work draws from the previous literature on the control of organo-metallic skeleton size [33] and proposes a method involving the batch additions of Fe(acac)_3_ to differentiate the nucleation and growth processes of IONs. This method aims to increase the size of the IONs modified by PEG and PEI. In this approach, the nucleation and growth of crystals are separated by gradually adding reaction materials in batches. Initially, nucleation occurs at a high supersaturation level, but as the monomer concentration decreases, nucleation ceases. The subsequent addition of an appropriate amount of Fe(acac)_3_ maintains the monomer concentration, allowing for crystal growth without promoting new nuclei formation. The nanoparticles prepared using this method exhibit significantly larger sizes than those synthesized via one-time addition of the reaction material, resulting in enhanced magnetic properties while still retaining good water dispersibility. These IONs can find applications in magnetic separation and other fields. This method of the batch additions of the reactant to increase the particle sizes may be used for other material systems.

## 2. Experiments

### 2.1. Materials and Reagents

Iron (III) acetylacetonate was purchased from Tokyo Kasei Kogyo Co., Ltd. (Tokyo, Japan). Poly (ethylene glycol) (PEG, Mw = 1000, 95%), toluene (99%) and acetone (99%) were purchased from Xilong Science Co., Ltd. (Shantou, China). Polyethyleneimine (PEI, Mw = 1800, 99%) was purchased from Shanghai Aladdin Biochemical Science Technology Co., Ltd. (Shanghai, China).

### 2.2. Preparation of IONs

PEG and PEI were used as solvents and modifiers to prepare IONs by decomposing Fe(acac)_3_ at a high temperature, respectively. The experimental procedure proceeded as follows: PEG and PEI were mixed in a three-necked flask at room temperature; subsequently, when the solution reached 80 °C, a portion of Fe(acac)_3_ was added, and the temperature was maintained at 80 °C for 10 min before being gradually increased to 320 °C at a rate of 10 °C/min. Throughout the process, the solution was stirred continuously and argon gas was introduced into the reaction system to eliminate dissolved oxygen and prevent the formation of by-products. The experimental design was as follows:

In the experimental (batch additions of the reactant) group, 0.7 g of Fe(acac)_3_ were added to the PEG and PEI at 80 °C. After holding at 320 °C for 60 min, 0.15 g of Fe(acac)_3_ were added 10 times at 12-minute intervals. The total amount of Fe(acac)_3_ added was 2.2 g and the total holding time was 180 min. The schematic diagram of the synthesis steps in the experiment group is as follows:
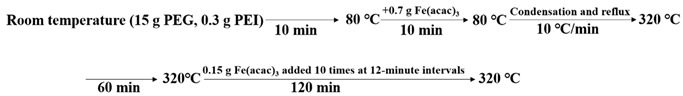


In the control (one-time addition of the reactant) group, 2.2 g of Fe(acac)_3_ were added one time to the PEG and PEI at 80 °C. The temperature was then raised to 320 °C and held for 180 min. The schematic diagram of the synthesis steps in the control group is as follows:
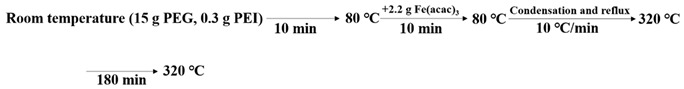


### 2.3. Characterization

X-ray diffraction (XRD, X’ Pert PRO, Amsterdam, The Netherlands) was used to analyze the crystal structure of the nanoparticles and determine the physical phase. Fourier transform infrared (FT-IR, NEXUS 670, Madison, WI, USA) spectroscopy was used to analyze the modification of organic matter on the surface of the magnetic nanoparticles. Malvern laser particle sizer (Nano ZS, Manchester, UK) was used to test the hydrodynamic particle size and zeta potential of particles in aqueous solution, which were used for observing the size distribution of the particles and the colloidal stability. Transmission electron microscope (TEM, FEI Tecnai F20, Hillsboro, OR, USA) was used to observe the magnetic nuclei of the magnetic nanoparticle size; the diameter size of the magnetic nuclei was counted, and the distribution range was analyzed using ImageJ software (ImageJ 1). The observation of the crystal structure of the samples was performed by using high-resolution transmission electron microscopy (HRTEM, FEI Tecnai F20, Hillsboro, OR, USA). A thermogravimetric analyzer (TGA, STA449, Stuttgart, German) was used to calculate the mass of the samples with water and organic modification layers being removed. The M-H curves of the nanoparticles obtained by different methods of syntheses were compared using a Magnetic Property Measurement System (MPMS, MPMS-3, San Diego, CA, USA) to evaluate their magnetic response properties.

## 3. Results and Discussion

### 3.1. X-ray Diffraction and Fourier Transform Infrared Spectroscopy of IONs

**Figure 1:**
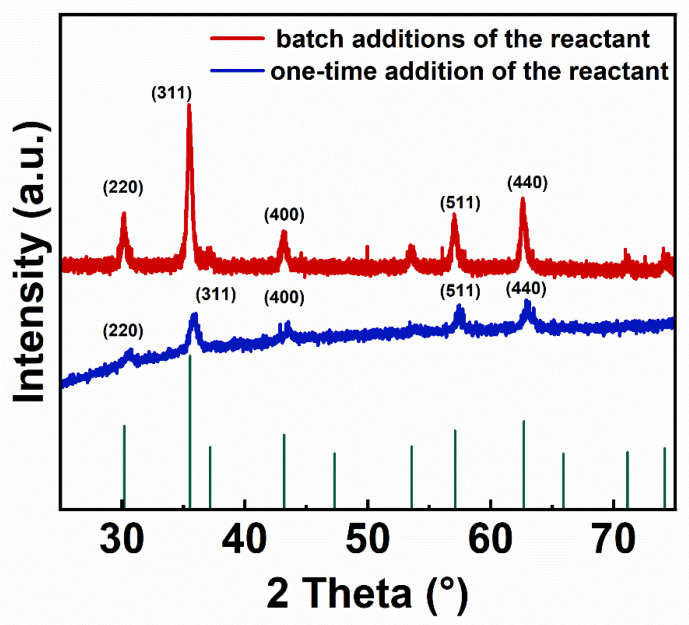
XRD patterns of IONs (iron oxide nanoparticles) prepared by the batch additions of the reactant (0.15 g Fe(acac)_3_ added 10 times at 12-minute intervals) and the one-time addition of the reactant (2.2 g Fe(acac)_3_ added at once during the initial stage of the reaction). The diffraction peaks correspond to the crystal planes of Fe_3_O_4_.

Figure 1 compares the XRD patterns of the IONs prepared in the batch additions of the reactant and one-time addition of the reactant. The wavelength of the X-rays used for testing is 0.154056 nm. The diffraction peaks of both samples have the same position, and the peak intensity distributions are consistent. This indicates that the crystal structure of the samples obtained by changing the synthesis method has not been altered. The diffraction peaks at 2θ values of 30.12°, 35.48°, 43.12°, 57.03° and 62.63° are consistent with the peak positions of the PDF standard card (75-0033) of Fe_3_O_4_, which, respectively, correspond to the (220), (311), (400), (511) and (440) crystal planes of Fe_3_O_4_, indicating that the prepared samples contain the Fe_3_O_4_ phase. By using the Scherrer formula (Formula (1)), the particle sizes of two ION products were calculated. In the formula, D represents the particle size of IONs. The shape factor k is set to 0.9. The wavelength of the incident radiation λ is 0.154056 nm. β represents the full width at half maximum (FWHM) of the diffraction peak corresponding to the (311) crystal plane (θ = 17.74°), which shows the strongest diffraction peak with values of 0.2598° and 0.5117° for IONs from the experimental group and the control group, respectively. The calculated particle sizes of IONs prepared by the batch additions of the reactant and by the one-time addition of the reactant were 32.1 nm, 16.3 nm, respectively. Thus, the particle size of IONs prepared by the batch additions of the reactant was significantly larger than that of IONs prepared by the one-time addition of the reactant.

(1) $$D=\frac{k\lambda }{\beta cos\theta }$$

**Figure 2:**
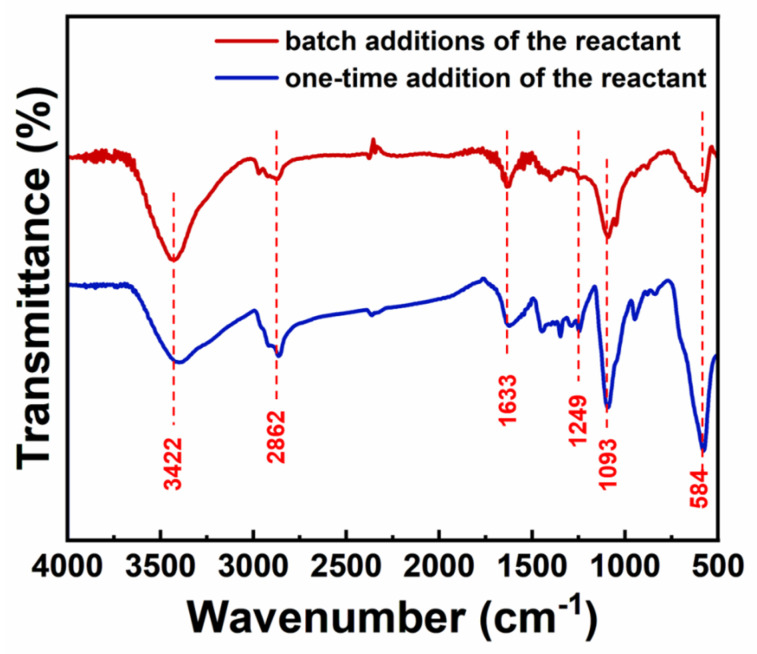
FTIR of IONs prepared by the batch additions of the reactant and the one-time addition of the reactant.

Figure 2 displays the nearly identical infrared absorption spectra of the IONs prepared by the two synthesis methods. During the reaction process of preparing the IONs, PEG was used as a reaction solvent, PEI as a modifier, and Fe(acac)_3_ as a precursor. The -OH group at the end of PEG was oxidized to -COOH [23], which was attached to the surface of nanoparticles through the covalent bonding of Fe-O, while PEI was bound to the nanoparticles through -NH_2_. Additionally, PEG and PEI may also be connected to each other through hydrogen bonding. The absorption peak at wavenumber 584 cm^−1^ corresponds to the characteristic peak of Fe-O. The absorption peak at 1093 cm^−1^ is attributed to the stretching vibration of the C-O bond. The absorption peak at 1249 cm^−1^ is attributed to the stretching vibration of amide III bond C-N [34]. The absorption peak at 1633 cm^−1^ is attributed to the symmetric stretching vibration of the -COO- group. The absorption peak at 2862 cm^−1^ represents the C-H characteristic absorption peak, and the absorption peak at 3422 cm^−1^ corresponds to -OH. The infrared absorption spectra of the samples indicate that the surface of the IONs prepared by the two methods is enriched with -OH and -COOH groups. The organic layer is successfully modified on the surface of the nanoparticles.

### 3.2. Analysis of Colloidal Stability and Particle Size Distribution of IONs

**Figure 3:**
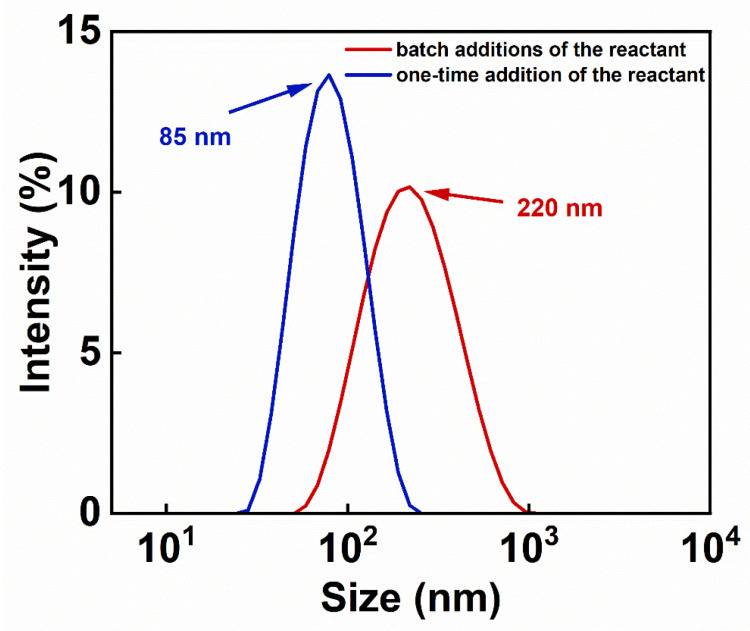
Hydrodynamic diameter distributions of IONs prepared by the batch additions of the reactant and the one-time addition of the reactant.

**Figure 4:**
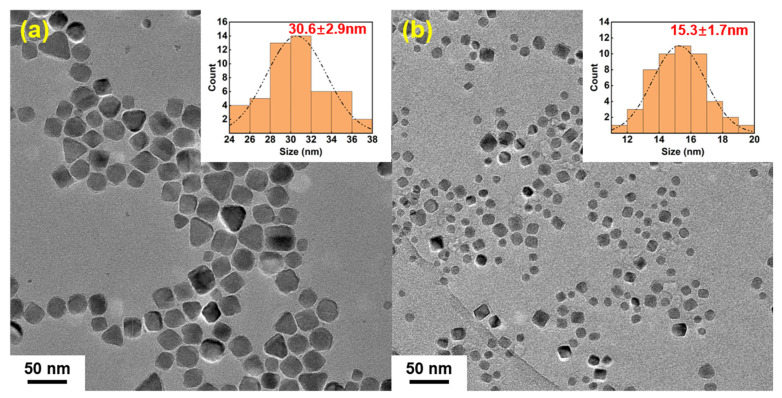
TEM image of IONs prepared by the batch additions of the reactant (**a**) and the one-time addition of the reactant (**b**); the histogram of particle size distribution with log-normal functions is shown as the inset.

**Figure 5:**
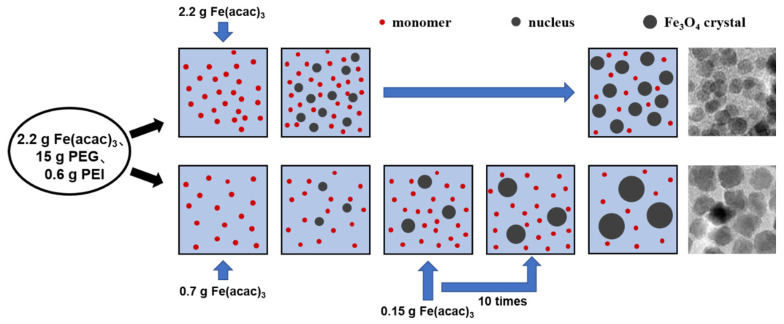
Schematic for the nucleation and growth process of IONs during the reactions of the one-time addition of the reactant (**top**) and the batch additions of the reactant (**bottom**). The TEM images of the two kinds of IONs prepared are shown on the right side of the schematic.

Dynamic light scattering (DLS) determines particle size by measuring the velocity of the Brownian motion of the particles in solution, which includes the size of the iron oxide crystals, the thickness of the organic layer modified on the surface, and the hydration layer. The organic layer on the IONs consists of PEG and PEI; PEG is a non-toxic hydrophilic solvent that is covalently bonded to IONs through Fe-O bonds. PEI is a water-soluble polymer that forms hydrogen bonds with both IONs and PEG. The modification of PEI and PEG enhances the water solubility of IONs. It can be found, in Figure 3, that the hydrodynamic sizes of the samples prepared in the batch additions of the reactant and the one-time addition of the reactant during the reaction were distributed around 220 nm and 85 nm, respectively. The particle size distribution of the IONs prepared by the batch additions of the reactant is relatively wide (Figure 3), which is consistent with the results of particle size statistics in the TEM images in the following Figure 4. In the process of the batch additions of the reactant, although the reactant added later is used mainly for crystal growth due to its lower concentration, there are still fewer nucleation processes occurring at this time (Figure 5). This increases the particle size distribution, and therefore, the hydrodynamic size distribution. The synthesis by the one-time addition of the reactant firstly produces a larger number of nuclei, but later produces few new nuclei, thus forming a relatively narrow size distribution. IONs prepared by batch addition of the reactant have PDI values below 0.3, which can be considered as still possessing good aqueous dispersibility.

**Figure 6:**
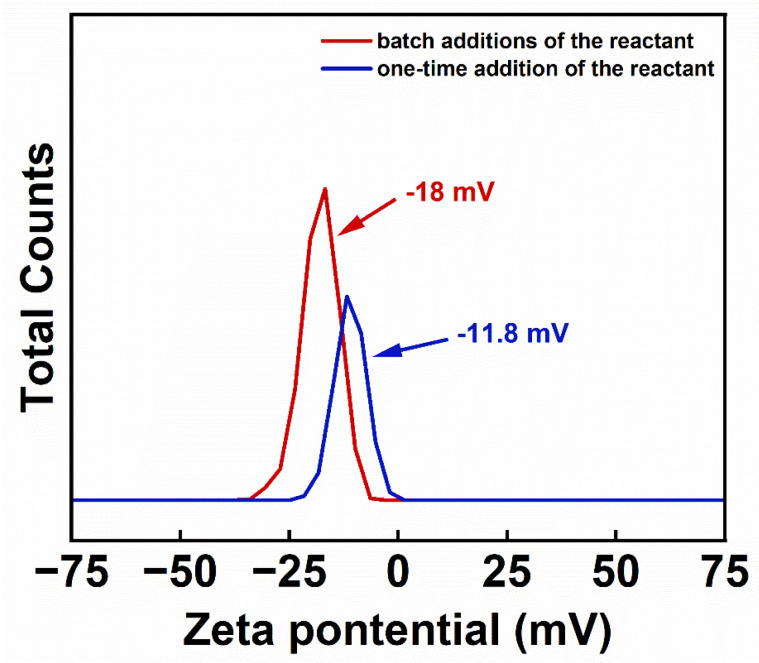
The zeta potential of the IONs prepared by the batch additions of the reactant and the one-time addition of the reactant.

Figure 6 shows the results of the zeta potential test of the aqueous solutions of IONs obtained by the two methods. The pH of the sample solutions was calibrated to 7.0 prior to testing. The zeta potential value of the sample solution prepared by the batch additions of the reactant and by the one-time addition of the reactant are −18 mV, −11.8 mV, respectively. The negative zeta potential may be due to the high concentration of Fe(acac)_3_, which results in more IONs with relatively few PEI modifications. The larger the absolute value of the zeta potential, the better the colloidal stability of the solution. By comparison, the sample prepared by the batch additions of the reactant exhibits better dispersion stability in aqueous solution.

**Figure 7:**
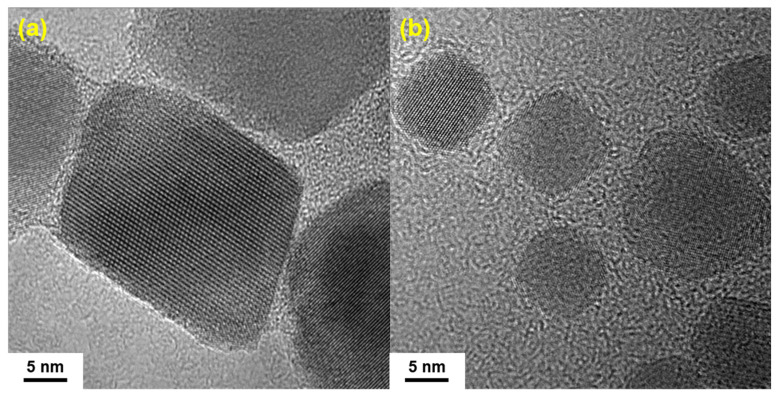
(**a**) HRTEM of IONs prepared by the batch additions of the reactant; (**b**) HRTEM of IONs prepared by one-time addition of the reactant.

As shown in Figure 4a,b, the morphology and sizes of the two samples were observed under TEM, and the diameters of nanoparticles were measured to calculate their size distribution. The diameter distributions of the IONs prepared by the batch additions and the one-time addition of Fe(acac)_3_ were 30.6 ± 2.9 nm, 15.3 ± 1.7 nm, respectively. The diameter of the IONs prepared by the batch additions of the reactant group were significantly larger than that prepared by the one-time addition of the reactant, which demonstrated that the batch additions of the reactant method that separate crystal nucleation from growth by adding the reactant step by step in the course of the reaction could effectively increase the size of the prepared nanoparticles. In addition, the HRTEM of the products in Figure 7a,b shows that the IONs prepared by the gradual addition of the Fe(acac)_3_ retain their highly crystalline nature.

The formation of nuclei and crystal growth means the transformation from a disordered state to an ordered state that is a first-order phase transition [35]. Certain energy barriers must be overcome during the formation of nuclei [36], and nucleation is more likely to occur when the supersaturation of a solution rises as the solute concentration increases. Figure 5 simulates the nucleation growth process in both the one-time addition of the reactant group and the batch additions of the reactant group. At the initial stage of the reaction, a larger number of nuclei can be formed in the one-time addition of the reactant group due to the addition of a larger amount of Fe(acac)_3_ because of the higher solution concentration and higher degree of supersaturation. The reactant in the solution diffuses toward the surface of the nuclei, and the growth units merge and crystals grow to form iron oxide nanocrystals of about 15.3 nm in size. In contrast, in the batch additions of the reactant group, the lower initial concentration leads to the generation of fewer nuclei. Subsequently, the addition of 0.15 g of Fe(acac)_3_ for 10 times is used to maintain the concentration of growth units in the reaction system for crystal growth. The reactant concentration in the solution is kept below the threshold favoring the overcoming of energy barriers for nucleus formation, resulting in few new nuclei being generated. Consequently, the initially formed nuclei eventually grow into larger IONs, with particle sizes of about 30.6 nm in our case. It should be noted that the particle size increase of nanoparticles simply by increasing the amount of the reactant, reaction time and temperature, but with the one-time addition of the reactant, is difficult to achieve at this level. It can be expected that this method of the batch additions of the reactant to increase the particle sizes may be used for other material systems.

### 3.3. Hysteresis Loop Analysis of IONs

**Figure 8:**
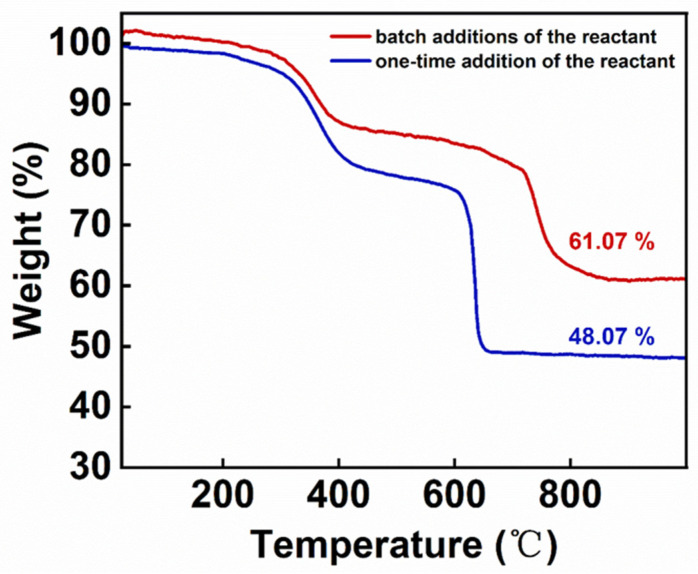
Thermogravimetric curves of IONs prepared by the batch additions of the reactant and the one-time addition of the reactant, tested under N_2_ atmosphere with a heating rate of 10 °C/min.

**Figure 9:**
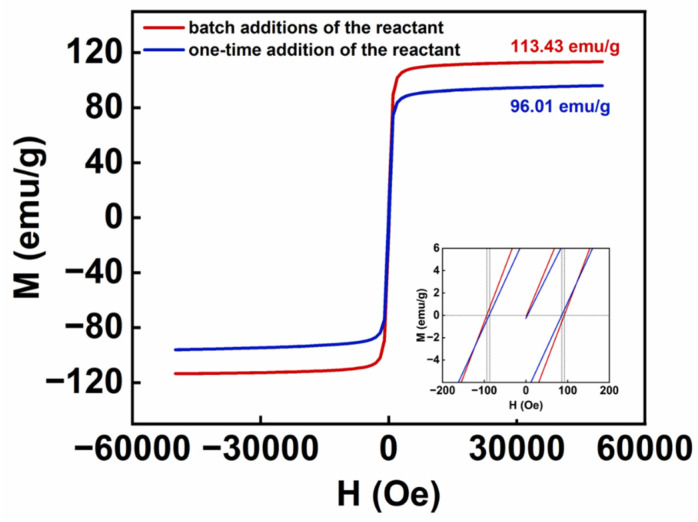
Hysteresis loop of IONs prepared by the batch additions of the reactant and the one-time addition of the reactant at 300 K. The inset presents the values of magnetic field strength H applied until the magnetization M = 0, which is used to analyze the intrinsic coercivity of the product.

The surface organic modifier content was calculated from the thermogravimetric curves, and the hysteresis loops of the two samples were analyzed after removing the mass of the organic modification layer. The thermogravimetric curves of the samples were tested under N_2_ atmosphere with a heating rate of 10 °C/min. In Figure 8, the thermogravimetric curve shows that the IONs prepared by the batch additions of the reactant contained less organic substance for surface modification; this is because, compared to the IONs obtained by the one-time addition of the reactant, the IONs prepared by the batch additions of the reactant are larger in size and have a smaller specific surface area. In Figure 9, the saturation magnetization at 300 K of the sample by the batch additions of the reactant and by the one-time addition of the reactant are 113 emu/g with an intrinsic coercivity of −94 Oe, and 96 emu/g with the intrinsic coercivity of −87 Oe, respectively. The results show that the sample prepared by the batch additions of the reactant in the reaction process possesses a higher saturation magnetization; this is because the saturation magnetization of magnetic nanoparticles generally increases with increasing particle size [21,26]. IONs prepared by the batch additions of the reactant have larger particle sizes, resulting in higher saturation magnetization.

Previous studies have utilized the co-precipitation method to synthesize magnetite nanoparticles coated with polyethyleneimine (PEI-Fe_3_O_4_) at 90 °C and 20 °C. The saturation magnetizations at 300 K of these nanoparticles are about 70 emu/g [37] and 60 emu/g [38], respectively. In contrast, the IONs prepared by the batch additions of the reactant in this work have a much higher saturation magnetization, indicating a better response to an external magnetic field.

The yield of IONs prepared by the thermal decomposition method can meet the demands in the field of medical research applications. The commercialization of the product can be achieved through increasing the reaction quantity and implementing production automation. These large particles can be used for the separation of cells, proteins, and nucleic acids, as well as for magnetic resonance imaging applications that require high imaging effects. 

## 4. Conclusions

In this work, we demonstrated a method to increase the size of the IONs by the batch additions of the reactant; this increase is due to the separation of nucleation and crystal growth. Specifically, the IONs prepared by the batch additions of the reactant have a particle size of 30.6 nm, with a saturation magnetization of 113 emu/g. These values are much greater than the particle size of 15.3 nm and the saturation magnetization of 96 emu/g observed in IONs prepared with the same amount of reactants and the same reaction time, but with the reactant added all at once. The IONs prepared by the batch additions still have good single crystal structure of the Fe_3_O_4_ phase and possess water-dispersion properties. Compared to the IONs prepared by the previously reported methods, such as thermal decomposition using PEG as a reaction solvent or co-precipitation, the IONs prepared by the batch additions of the reactant in this work exhibited the largest particle size and the highest magnetization. These IONs are expected to have better applications in biomedical fields such as magnetic separation. This method of the batch additions of the reactant to increase particle size may be used for other material systems.

## Data Availability

Data are contained within the article.

## References

[1] Awogbemi O., Von Kallon D.V. (2024). Recent advances in the application of nanomaterials for improved biodiesel, biogas, biohydrogen, and bioethanol production. Fuel.

[2] Javed R., Zia M., Naz S., Aisida S.O., Ul Ain N., Ao Q. (2020). Role of capping agents in the application of nanoparticles in biomedicine and environmental remediation: Recent trends and future prospects. J. Nanobiotechnol..

[3] Zhao X., Xuan J., Li Q., Gao F., Xun X., Liao Q., Zhang Y. (2023). Roles of Low-Dimensional Nanomaterials in Pursuing Human-Machine-Thing Natural Interaction. Adv. Mater..

[4] Joseph T.M., Mahapatra D.K., Esmaeili A., Piszczyk L., Hasanin M.S., Kattali M., Haponiuk J., Thomas S. (2023). Nanoparticles: Taking a Unique Position in Medicine. Nanomaterials.

[5] Sharma P., Otto M. (2024). Multifunctional nanocomposites modulating the tumor microenvironment for enhanced cancer immunotherapy. Bioact. Mater..

[6] Shi Y., Gao F., Zhang Q., Yang J. (2023). Covalent Organic Frameworks: Recent Progress in Biomedical Applications. ACS Nano.

[7] Zhang M., Xu F., Cao J., Dou Q., Wang J., Wang J., Yang L., Chen W. (2024). Research advances of nanomaterials for the acceleration of fracture healing. Bioact. Mater..

[8] Zhou Z., Wang X., Zhang H., Huang H., Sun L., Ma L., Du Y., Pei C., Zhang Q., Li H. (2021). Activating Layered Metal Oxide Nanomaterials via Structural Engineering as Biodegradable Nanoagents for Photothermal Cancer Therapy. Small.

[9] Pankhurst Q.A., Connolly J., Jones S.K., Dobson J. (2003). Applications of magnetic nanoparticles in biomedicine. J. Phys. D Appl. Phys..

[10] Xu P.A., Zeng G.M., Huang D.L., Feng C.L., Hu S., Zhao M.H., Lai C., Wei Z., Huang C., Xie G.X. (2012). Use of iron oxide nanomaterials in wastewater treatment: A review. Sci. Total Environ..

[11] Dadfar S.M., Roemhild K., Drude N.I., von Stillfried S., Knüchel R., Kiessling F., Lammers T. (2019). Iron oxide nanoparticles: Diagnostic, therapeutic and theranostic applications. Adv. Drug Deliv. Rev..

[12] Abuawad A., Ashhab Y., Offenhaeusser A., Krause H.-J. (2023). DNA Sensor for the Detection of Brucella spp. Based on Magnetic Nanoparticle Markers. Int. J. Mol. Sci..

[13] Luther D.C., Huang R., Jeon T., Zhang X.Z., Lee Y.W., Nagaraj H., Rotello V.M. (2020). Delivery of drugs, proteins, and nucleic acids using inorganic nanoparticles. Adv. Drug Deliv. Rev..

[14] Aghebati-Maleki A., Dolati S., Ahmadi M., Baghbanzhadeh A., Asadi M., Fotouhi A., Yousefi M., Aghebati-Maleki L. (2020). Nanoparticles and cancer therapy: Perspectives for application of nanoparticles in the treatment of cancers. J. Cell. Physiol..

[15] Fahmy S.A., Alawak M., Brüssler J., Bakowsky U., El Sayed M.M.H. (2019). Nanoenabled Bioseparations: Current Developments and Future Prospects. Biomed. Res. Int..

[16] Krasitskaya V.V., Kudryavtsev A.N., Yaroslavtsev R.N., Velikanov D.A., Bayukov O.A., Gerasimova Y.V., Stolyar S.V., Frank L.A. (2022). Starch-Coated Magnetic Iron Oxide Nanoparticles for Affinity Purification of Recombinant Proteins. Int. J. Mol. Sci..

[17] Schwaminger S.P., Fehn S., Steegmüller T., Rauwolf S., Löwe H., Pflüger-Grau K., Berensmeier S. (2021). Immobilization of PETase enzymes on magnetic iron oxide nanoparticles for the decomposition of microplastic PET. Nanoscale Adv..

[18] Altalbawy F.M.A., Ali E., Fenjan M.N., Mustafa Y.F., Mansouri S., Bokov D.O., Idiyevna S.G., Misra N., Alawadi A.H., Alsalamy A. (2024). Aptamer-Magnetic Nanoparticle Complexes for Powerful Biosensing: A Comprehensive Review. Crit. Rev. Anal. Chem..

[19] Wang Y., Yang H. (2009). Synthesis of iron oxide nanorods and nanocubes in an imidazolium ionic liquid. Chem. Eng. J..

[20] Zhang R.R., Lu K., Xiao L., Hu X.L., Cai W., Liu L.J., Liu Y., Li W.H., Zhou H., Qian Z.Y. (2023). Exploring atherosclerosis imaging with contrast-enhanced MRI using PEGylated ultrasmall iron oxide nanoparticles. Front. Bioeng. Biotech..

[21] Sun S.H., Zeng H., Robinson D.B., Raoux S., Rice P.M., Wang S.X., Li G.X. (2004). Monodisperse MFe_2_O_4_ (M = Fe, Co, Mn) nanoparticles. J. Am. Chem. Soc..

[22] Shi D., Beasock D., Fessler A., Szebeni J., Ljubimova J.Y., Afonin K.A., Dobrovolskaia M.A. (2022). To PEGylate or not to PEGylate: Immunological properties of nanomedicine’s most popular component, polyethylene glycol and its alternatives. Adv. Drug Deliv. Rev..

[23] Yan X.J., Han G.H., Wang S., Chong C.G., Han D., Tan J., Zhang B.L. (2021). The distribution of the iron oxide nanoparticles modified with polyethylene glycol in rat brains. Mater. Chem. Phys..

[24] Wu W., Wu Z.H., Yu T., Jiang C.Z., Kim W.S. (2015). Recent progress on magnetic iron oxide nanoparticles: Synthesis, surface functional strategies and biomedical applications. Sci. Technol. Adv. Mat..

[25] Hufschmid R., Arami H., Ferguson R.M., Gonzales M., Teeman E., Brush L.N., Browning N.D., Krishnan K.M. (2015). Synthesis of phase-pure and monodisperse iron oxide nanoparticles by thermal decomposition. Nanoscale.

[26] Tan Y.W., Zhuang Z.B., Peng Q., Li Y.D. (2008). Room-temperature soft magnetic iron oxide nanocrystals: Synthesis, characterization, and size-dependent magnetic properties. Chem. Mater..

[27] Park J., An K.J., Hwang Y.S., Park J.G., Noh H.J., Kim J.Y., Park J.H., Hwang N.M., Hyeon T. (2004). Ultra-large-scale syntheses of monodisperse nanocrystals. Nat. Mater..

[28] Han D., Zhang B.L., Su L.C., Han G.H., Wang S. (2019). Synthesis of superparamagnetic iron oxide nanoparticles with different particle sizes and its magneto-calorific effects under alternating current magnetic field. J. Mater. Eng..

[29] Erdemir D., Lee A.Y., Myerson A.S. (2009). Nucleation of Crystals from Solution: Classical and Two-Step Models. Acc. Chem. Res..

[30] LaMer V.K., Dinegar R.H. (1950). Theory, production and mechanism of formation of monodispersed hydrosols. J. Am. Chem. Soc..

[31] Lee J., Yang J., Kwon S.G., Hyeon T. (2016). Nonclassical nucleation and growth of inorganic nanoparticles. Nat. Rev. Mater..

[32] Liu H., Zhao Y., Sun J. (2023). Heterogeneous Nucleation in Protein Crystallization. Biomimetics.

[33] Lan X., Huang N., Wang J., Wang T. (2018). A general and facile strategy for precisely controlling the crystal size of monodispersed metal-organic frameworks via separating the nucleation and growth. Chem. Commun..

[34] Wang S., Zhang B.L., Su L.C., Nie W., Han D., Han G.H., Zhang H., Chong C.G., Tan J. (2019). Subcellular distributions of iron oxide nanoparticles in rat brains affected by different surface modifications. J. Biomed. Mater. Res. A.

[35] Sosso G.C., Chen J., Cox S.J., Fitzner M., Pedevilla P., Zen A., Michaelides A. (2016). Crystal Nucleation in Liquids: Open Questions and Future Challenges in Molecular Dynamics Simulations. Chem. Rev..

[36] Kwon S.G., Hyeon T. (2011). Formation Mechanisms of Uniform Nanocrystals via Hot-Injection and Heat-Up Methods. Small.

[37] Leon Felix L., Rodriguez Martinez M.A., Pacheco Salazar D.G., Huamani Coaquira J.A. (2020). One-step synthesis of polyethyleneimine-coated magnetite nanoparticles and their structural, magnetic and power absorption study. RSC Adv..

[38] Gerulova K., Kucmanova A., Sanny Z., Garaiova Z., Seiler E., Caplovicova M., Caplovic Ľ., Palcut M. (2022). Fe_3_O_4_-PEI Nanocomposites for Magnetic Harvesting of Chlorella vulgaris, Chlorella ellipsoidea, Microcystis aeruginosa, and Auxenochlorella protothecoides. Nanomaterials.

